# Effects of multi-directional step exercise with weight-shifting as an adjunct to conventional exercises on balance and gait in stroke patients

**DOI:** 10.1038/s41598-022-21073-y

**Published:** 2022-10-12

**Authors:** Rakesh Shrestha, T. S. Sandesh, Zainab Jalal, Shibili Nuhmani, Ahmad H. Alghadir, Masood Khan

**Affiliations:** 1 Alva’s College of Physiotherapy, Moodbidri, Karnataka India; 2 New Horizons Child Development Centre, Mumbai, Maharashtra India; 3 Department of Physical Therapy, College of Applied Medical Sciences, Imam Abdulrahman Bin Faisal University, Dammam, Saudi Arabia; 4 Rehabilitation Research Chair, Department of Rehabilitation Sciences, College of Applied Medical Sciences, King Saud University, Riyadh, Saudi Arabia

**Keywords:** Stroke, Rehabilitation

## Abstract

Stroke patients have gait dysfunctions that affect their activities of daily living. Stroke patients should be able to take multi-directional steps as it is necessary to achieve an independent gait. The study aimed to examine the effects of multi-directional step exercises (MSE) along with weight-shifting as an adjunct to conventional therapeutic exercises (CTE) on functional gait performance and balance in patients with stroke. Twenty-four stroke patients (mean age 56.75 years) participated in the study and were divided into experimental and control groups. The experimental group (EG) included MSE along with weight shifting and CTE. The control group (CG) included only CTE. Treatment intervention lasted for 4 weeks. Gait and balance were measured using the functional gait assessment (FGA) and the berg balance scale (BBS), respectively. EG showed a significant improvement (p = 0.000) in both the BBS and FGA scores. In CG, a significant improvement (p = 0.000) was observed only in FGA scores. EG showed a greater improvement in scores of BBS (p = 0.000) and FGA (p = 0.000) than CG. Four weeks of MSE in conjunction with CTE were more effective in improving balance and functional gait performance compared to CTE alone in the selected stroke population.

## Introduction

Stroke is one of the major causes of permanent disability^1^. Stroke can have many causes and occurs mainly in old age, although it can occur at any age^2^. Most stroke survivors can perform limited activities of daily living (ADL) due to motor, sensory, emotional, and cognitive impairments^3^. Stroke patients commonly exhibit symptoms of hemiplegia and thus, as a result, have defects in voluntary movements, asymmetrical weight-shifts, gait, and body balance^4^. Stroke patients have decreased balance ability due to their centre of gravity shifting towards the unaffected side^4^. In the standing posture, approximately 61% to 80% of their body weight is concentrated on the unaffected lower extremity^5^. Due to these asymmetric postures, stroke patients may have frequent falls, limited independent gait, and reduced gait velocity^6^. This asymmetric gait in hemiplegic patients may cause reduced bone density on the affected side and an overall increase in energy expenditure on walking^7,8^. In addition to normal walking, stroke survivors fall during transfers between beds and the wheelchair and during standing turns^9,10^. These gait dysfunctions after stroke are the main cause of impaired functional ambulation, which in turn causes decreased social participation and poor quality of life. Therefore, enhancing the tolerance and capability to bear weight on the affected lower extremity and achieving good body balance during various functional activities and symmetric gait patterns are the main goals of rehabilitation for stroke patients^11–13^.

Multi-directional steps are essential for ADL because, while walking indoors, 35–50% of all steps consist of those steps that change the direction of movement, i.e. non-straight steps^14^. Normal individuals and stroke patients should have the ability to take steps in multiple directions to perform ADL safely. In previous studies, step training in various directions has been reported to improve balance and gait in older people and patients with Parkinson’s disease^15,16^. The stroke patients also have impaired ability to bear weight on the paretic side in all three directions of a step, i.e. forward, backward and lateral^17^. Therefore, a rehabilitation program for stroke survivors should include multi-directional step exercises (MSE) and weight shifting training. One previous study by Park et al.^4^ compared the multi-directional stepping training versus general physical therapy in stroke patients. Park et al. used two stair-shaped footholds (10 cm) and a weight rod for multi-directional stepping training. They reported improvement in the berg balance scale (BBS), timed up and go (TUG) test, 10-m walk test, and fall efficacy scores after performing multi-directional stepping training compared to general physical therapy. In their study, participants performed stepping exercises over stair-shaped footholds and across the weight rod by the non-paretic side while keeping the paretic side fixed over the ground.

It will be interesting to examine the effects of MSE along with weight-shifting when performed by the paretic side of the participants while keeping the non-paretic side fixed over the ground. To the best of our knowledge, no study has examined these effects in stroke patients. Since it will be difficult for participants to take steps over a foothold by their paretic side, therefore, in the present study these exercises will be performed on the flat surface. Thus, this study was conceptualized to examine the effects of MSE along with weight-shifting when performed as an adjunct to conventional therapeutic exercises (CTE), on balance and functional gait performance in patients with stroke. We hypothesized that the addition of MSE along with weight-shifting to a 4-week program of CTE improves balance and functional gait performance in comparison to CTE alone in stroke patients.

## Materials and methods

### Participants' eligibility and ethical clearance

The study's sample size was calculated using the software G*Power^18^, version 3.1.9.4 (https://www.psychologie.hhu.de/arbeitsgruppen/allgemeine-psychologie-und-arbeitspsychologie/gpower). Mean difference, standard deviation, and effect size values were taken for variable BBS scores from the study of Park et al.^4^. Power was set at 0.95, and using the option of a two-tailed test, this calculation was done, which revealed 12 as the minimum total sample size. Therefore, 12 participants in each group were decided for recruitment, which made the total sample size 24.

Twenty-four patients with stroke were selected from the inpatient department of the Alva’s Health Centre hospital, Moodbidri, the Alva’s Ayurveda hospital, Vidhyagiri, and the outpatient department of the Alva’s College of Physiotherapy. The stroke was diagnosed by CT or magnetic resonance imaging. The recruited participants also had to have Mini-Mental status examination score greater than 21, the ability to maintain an independent standing posture for 30 s, should be able to walk independently without the use of an assistive device for at least 3 m, and could communicate and understand the therapist’s verbal commands. An expert physical therapist performed these examinations.

Participants who had a history of neurological pathologies (eg Alzheimer’s or Parkinson’s disease), visual deficits/disorders, concomitant medical disease (recent myocardial infarction, unstable angina, ventricular tachycardia), impaired balance, such as vertigo, dementia, impaired alertness, lower motor neuron lesion, severe orthopedic disease of the lower extremities, taking antispasmodic medications (eg Baclofen, Dantrolene sodium, etc.) or medications affecting balance were excluded from the study.

Ethics clearance was obtained from the ethics committee of Alva’s college of Physiotherapy (ACP/OP/2016/OL 06). All methods were performed in accordance with the relevant guidelines and regulations. The study was retrospectively registered on the public trial registry (ClinicalTrials.gov PRS; Registration No. NCT05285241 on 17/03/2022). Before the inclusion of participants in the study, the study details were explained to them, and written informed consent was obtained.

### Study design, the protocol of the study, and randomization of participants

**Figure 1:**
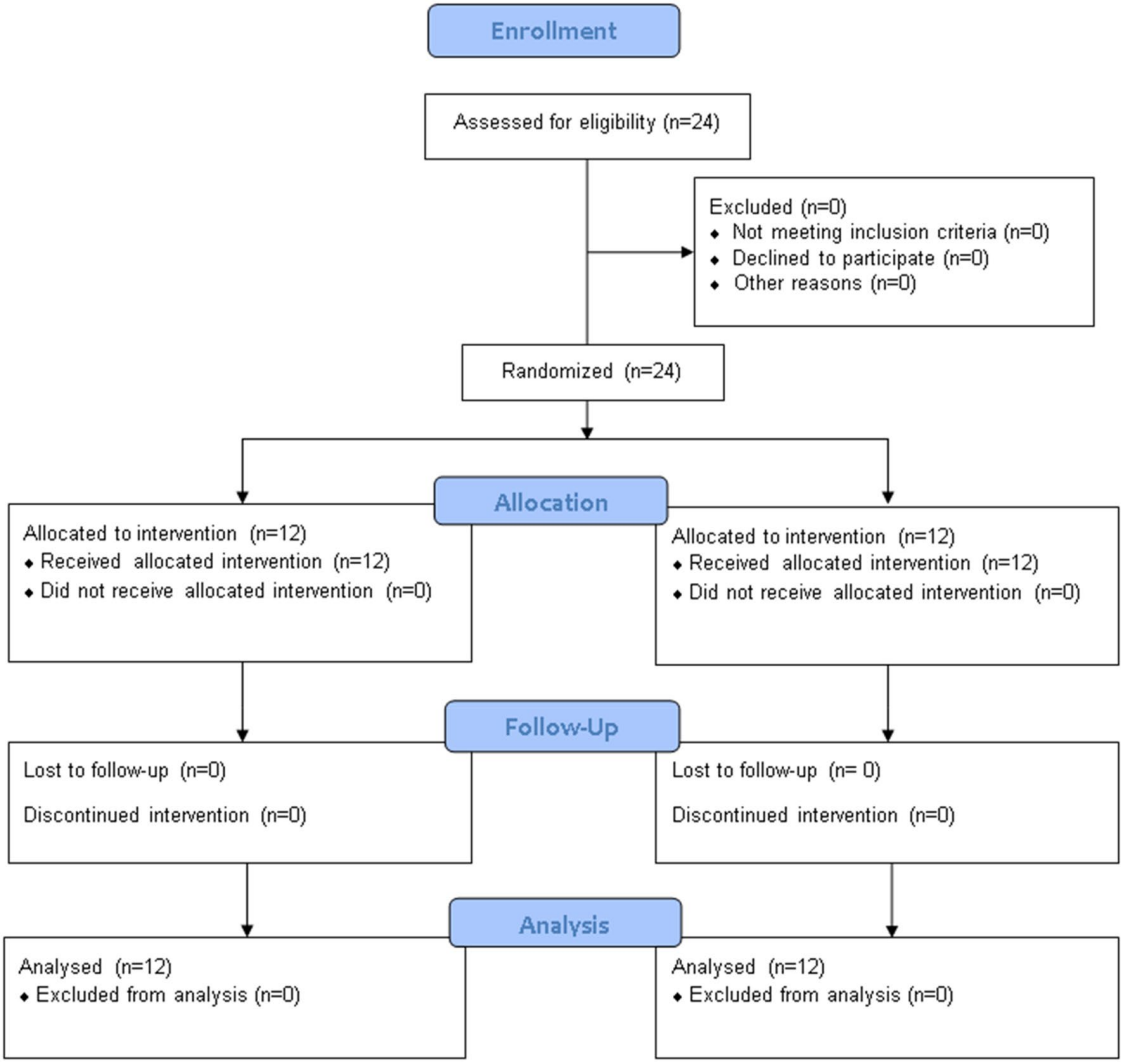
Consolidated Standards of Reporting Trials (CONSORT) flow chart of the study.

Two arms pretest–posttest experimental design was used. First, a detailed examination was performed that included the Mini-Mental State Examination (MMSE) to satisfy the inclusion and exclusion criteria. Two groups were formed: the experimental and the control group. The experimental group included MSE along with weight-shifting and CTE program while the control group included CTE program only. A physical therapy assistant, who did not measure the outcome measures, generated a random sequence and equally allotted the participants to two groups using the website randomization.com and lotteries. A total of 4 treatment sessions were given to each participant in both groups for a total of 4 weeks. The CTE sessions lasted approximately 45 min in both groups; however, in the experimental group, an additional 15 min was spent on MSE along with weight-shifting. Pre-test and post-test measurements of outcome measures were performed by the same physical therapist with 3 years of experience. The outcome assessor was kept blind to the allocation. Figure 1 includes the allocation and randomization of participants.

### Materials required

- 20-foot walkway
- Footstool or step
- Two standard chairs, one with an armrest and the other without
- Stairs with railing
- Foot stepper
- Stopwatch
- Yardstick
- Mirror

### Outcome measures

- Balance: measured by Berg Balance Scale (BBS)^19^.
- Functional gait performance: measured by Functional Gait Assessment (FGA)^20,21^.

BBS consists of 14 items and can be used to quantitatively monitor the progression of balance in stroke patients^19^. It has high intra- and inter-rater reliability and validity^22,23^. For BBS measurement, the outcome assessor asked the participants to perform 14 predetermined tasks which were graded from 0 to 4 according to their performance. Therefore, in BBS, minimum and maximum possible scores are 0 and 56, respectively^19^. The lower the score, the more impaired the balance will be.

FGA assesses the patient’s ability to perform multiple tasks and postural stability while walking. This tool is a modified version of the dynamic gait index, consists of 10 items, and has good reliability and validity for assessing walking balance in stroke patients who can ambulate independently^20,21^. For FGA measurement, the outcome assessor asked the participants to perform 10 predetermined tasks, and according to their performance, these tasks were graded from 0 to 3. Therefore, in FGA, the minimum and maximum possible scores are 0 and 30, respectively, where the higher score indicates better functional gait performance and health-related quality of life^24^.

The outcome assessor measured outcome measures before the start of intervention for baseline values and after 4-weeks of intervention.

### Interventions

Treatment intervention in both groups was performed for 4-weeks.

#### Experimental group

In this group, MSE and weight-shifting were performed along with CTE.

##### Conventional therapeutic exercises (CTE)

This included wall-supported gait training for around 10 min/session, functional mat exercises (supine to prone on the elbow, to prone on hand, to quadruped position, to kneeling, then at the end to standing, each position was maintained for 2 min) for around 10 min/session, stretching exercises (for hamstrings, quadriceps, gastrocnemius, glutei, elbow, and finger flexors, with 15–30 s hold for three repetitions for each muscle) for around 5 min/session, passive range of motion (ROM) exercises for the affected joints for about 10 min/session, active-assisted ROM exercises to progressive resistance exercises (PRE) with 10–15 s hold, 15 repetitions for affected muscles. Participants performed these exercises according to their abilities.

##### Multi-directional step exercises (MSE) along with weight-shifting

Participants were asked to stand barefoot with their hands on their sides. A mirror was placed in front of them for visual feedback. A physical therapy assistant stood beside them to support them in case of any balance disturbance. The first position is that stroke patients with hemiplegia have asymmetrical weight distribution between their lower extremities; therefore, they were asked to distribute their body weight between both lower extremities equally. In the second position, they had to take a sideway step with the affected lower extremity along with shifting their trunk towards the affected side, so that body weight passes through the affected foot. Then they had to return to the first position with an equal body weight distribution between both lower extremities. In the third position—they had to take a forward step with the affected lower extremity along with their trunk bending slightly forward so that the body weight passes through the forefoot of the affected side. Then they had to return to the first position. In the fourth position, they had to take a backward step along with shifting the trunk backwards so that body weight passed through the heel of the affected side. Then again, they had to return to the first position. Every position was to be maintained for 10 s each, and after completion of one set, a 2-min rest was given. This process had been repeated a total of three times.

##### Walking with wall support

Participants were asked to wear comfortable shoes and stand near the wall with the affected side towards it. A 3 cm wide and 10-m-long line was drawn parallel to the wall (20 cm away). The participants had to place their affected foot on the line while walking. Participants were instructed to touch the wall with their shoulders by shifting their trunks during the stance phase of the affected side. This way, the participants walked along the wall 10 times in one set. A total of 3 sets were performed with a 2-min rest between the sets.

#### Control group

Participants in this group performed CTE similar to the experimental group. In this group, participants also performed these exercises according to their abilities. These included wall-supported gait training for around 10 min/session, functional mat exercises for about 10 min/session, stretching exercises for about 5 min/session, passive range of motion (ROM) exercises for the affected joints for about 10 min/session, and active-assisted ROM exercises to progressive resistance exercises (PRE) with 10–15 s hold, 15 repetitions for affected muscles.

### Data analysis

SPSS statistical software^25^, version 26 (SPSS Inc., Chicago, IL, USA), was used for statistical analysis (https://www.ibm.com/support/pages/downloading-ibm-spss-statistics-26). The distribution of baseline values of dependent variables was analyzed using the Shapiro–Wilk test of normality. The test showed a normal distribution for both groups' baseline values of BBS and FGA. Therefore, for further with-in and between-group analysis, parametric tests were used. With-in-group and between-group analyses were performed using the paired samples and independent samples test, respectively. The confidence interval was established at 95%, and p < 0.05 was considered significant.

## Results

Demographic data and the p-values of the Shapiro–Wilk test of normality are presented in Table 1. This test revealed normal distribution for pre-intervention values of both variables; therefore, parametric tests were used for further with-in and between-group analyses.

**Table 1:** Demographic data, mean values of dependent variables, and the p-values for the Shapiro–Wilk test of normality.

	Control	p-value	Experimental	p-value
**Age**				
Mean (years) ± SD	62.16 ± 8.88		51.33 ± 11.84	
Range (years)	48–79		33–75	
**Gender**				
Male (n)	6		6	
Female (n)	6		6	
Total number of participants	12		12	
**No. of participants according to the site of lesion**				
Right CVA				
Male/female	3/1		4/3	
Left CVA				
Male/female	3/5		2/3	
**No. of participants according to the age group**				
30–40	0		2	
41–50	1		2	
51–60	5		7	
61–70	4		0	
71–80	2		1	
BBS (pre-intervention) (mean ± SD)	43.83 ± 6.16	0.433	41.91 ± 4.85	0.647
BBS (post-intervention) (mean ± SD)	44.08 ± 6.21		50.83 ± 3.43	
FGA (pre-intervention) (mean ± SD)	14.00 ± 4.89	0.661	10.58 ± 3.34	0.825
FGA (post-intervention) (mean ± SD)	14.66 ± 4.83		20.66 ± 4.94	

BBS: Berg Balance Scale; FGA: functional gait assessment.

### With-in group analysis (paired samples test) (Table 2)

**Table 2:** With-in-group comparison results for dependent variables in both groups.

		Mean difference	SD	SEM	t	df	p-value
Control group	BBS post–BBS pre	0.25	0.45	0.13	1.91	11	0.082
	FGA post–FGA pre	0.66	0.88	0.25	2.60	11	0.025*
Experimental group	BBS post–BBS pre	8.91	2.99	0.86	10.30	11	0.000*
	FGA post–FGA pre	10.08	3.14	0.90	11.10	11	0.000*

SD: standard deviation; SEM: standard error mean; BBS: Berg Balance Scale; FGA: functional gait assessment.

*Significant.

#### Experimental group

For BBS: a significant improvement in the BBS scores was observed in the experimental group from 41.91 to 50.83 (p = 0.000).

For FGA: a significant improvement in the FGA scores was observed in the experimental group from 10.58 to 20.66 (p = 0.000).

#### Control group

For BBS: no significant difference was observed in BBS scores in the control group (from 43.83 to 44.08) (p = 0.082).

For FGA scores: a significant improvement was observed in the FGA scores in the control group from 14.00 to 14.66 (p = 0.025).

### Between-group analysis (independent samples test) (Table 3)

**Table 3:** Between-group comparison results for both variables.

	t	df	Std. Error Difference	Sig. (2-tailed)	Cohen’s d
BBS post–BBS pre	− 9.90	22	0.87	0.000*	4.041
FGA post–FGA pre	− 9.97	22	0.94	0.000*	4.073

BBS: Berg Balance Scale; FGA: functional gait assessment.

*Significant.

When both groups were compared, a significant difference was observed in the mean differences of both variables [BBS (p = 0.000) and FGA (p = 0.000)]. The experimental group (mean difference, BBS = 8.91; FGA = 10.08) showed a greater improvement in BBS and FGA scores than the control group (mean difference, BBS = 0.25; FGA = 0.66).

## Discussion

The present study aimed to evaluate the effects of MSE along with weight-shifting on balance and functional gait performance in stroke patients when used as an adjunct to a 4-week CTE program. The results indicated that MSE along with weight-shifting, when performed in addition to a 4-week CTE program, was more effective in improving balance (BBS score) and gait performance (FGA score) than a CTE program alone in the selected stroke population. Significant improvement in FGA scores was also observed with the CTE program alone. However, a greater improvement was observed for both BBS and FGA scores after performing MSE along with weight-shifting in conjunction with the CTE program.

A combination of cognitive, emotional, sensory, and motor impairments occurs in patients after stroke^26^. These impairments limit the ability of stroke survivors to perform basic ADL^3^. 80% of patients with early-stage stroke lose their walking ability and begin to recover in 6 months. Stroke survivors commonly show hemiplegia symptoms leading to asymmetric weight-shifts, gait and body balance, and impaired voluntary movements. Their ability to bear weight on the paretic limb is impaired^17^. In stroke patients, the paretic limb has reduced ability of weight-shifting in three directions of a step, i.e. forward, backward and lateral and particularly greater loss in the forward direction^17^. Weight-bearing by the lower limbs is necessary for normal functional mobility in various directions and positions^17^. All these impairments, along with muscle weakness and loss of coordination, lead to gait dysfunction and reduced balance ability^27^. All these dysfunctions lead to decreased gait velocity, hampered independent gait, and may lead to falls^6^. Therefore, in patients with hemiplegic stroke, they must be confident that the lower limb of the affected side are ready and trained to bear the full body weight and generate the necessary muscle force to match the speed of the unaffected side^28^. Therefore, a few main goals of post-stroke rehabilitation are to enhance the weight-bearing capability and tolerance of the paretic limb during different tasks and to enhance the gait symmetry and independent gait thereby improving stroke patients' functions and social activities^11,29^.

Along with weight-shifting, multi-directional steps are also essential to gait during ADL. Stroke survivors should be able to take multi-directional steps to perform routine daily tasks. Therefore, to improve the ability of stroke patients to take multi-directional steps, i.e. steps in forward, lateral and backward directions and to enhance the weight-shifting ability, the MSE along with weight-shifting was added to the CTE program in the present study. The CTE program included AROM exercises, strengthening exercises, functional mat exercises, stretching exercises, and gait training.

The improvement observed in the present study in balance and functional gait performance is not surprising because an association between balance and weight-shifting has been reported previously. Eng and Chu^17^ suggested that poor balance in stroke patients may be due to the impairment in the weight-shifting ability of their hemiparetic side. The study by Jung et al.^30^ reported improved trunk control, trunk proprioception and balance by weight-shifting training on an unstable surface in chronic stroke patients. Learning to load and unload the paretic limb while standing is a crucial stage in the gait and balance training of stroke patients because the ability to start and control the voluntary weight-shifts toward either limb is a requirement for independent gait^31^. These previous studies already indicated an association between weight-shifting ability and balance in stroke patients; therefore, the improvements obtained in the present study were expected. BBS and FGA consist of different tasks which are related to each other. Improvement in one task may result in improvement in another task. For example, a study by Winstein et al.^32^ reported improved weight-bearing symmetry in standing after weight-shifting exercises. Similar findings supporting the association of tasks were also reported by Dean and Shephard^33^. Therefore, in the present study, MSE along with weight-shifting combined with the CTE program may have improved the ability of the hemiparetic side to take steps in different directions and bear more weight. Therefore, these improvements may have improved other tasks of BBS and FGA, resulting in an overall increment in scores.

One previous study by Park et al.^4^ reported similar results. They used multi-directional stepping training for 6 weeks, using two footholds and a weight rod for the non-paretic side in stroke patients and used BBS, TUG, 10-m walk test, and falls efficacy scale scores as outcome measures. They reported improvement in all outcome measures in comparison to the control group. However, the practical applicability of Park et al.’s study is not similar to the present study as they used stepping exercises by the non-paretic side of the patients however, in the present study stepping exercises were performed by the paretic side of the patients.

Another recently performed study by Choi and Kim^34^ compared the combined effects of multi-directional step-up training along with rhythmic auditory stimulation versus the multi-directional step-up training alone in stroke patients. Their effects were assessed on gait and balance ability. They concluded that multi-directional step-up training alone or with rhythmic auditory stimulation could improve balance and gait in stroke patients. However, greater improvement can be achieved when multi-directional step-up training is accompanied by rhythmic auditory stimulation.

Most studies that have been conducted on the improvement of balance and functional gait performance in stroke population have used lavish equipment like force plate systems, gyroscopes etc. However, the exercises performed in the present study are extremely easy and convenient to follow up even in a community step-up without any use of appliances. Therefore, the physical therapist may consider adding MSE along with weight-shifting to the CTE program of stroke rehabilitation as it does not require sophisticated equipment and can easily be performed by stroke patients.

There is some scope for future studies and limitations in the present study. Firstly, stroke patients, irrespective of their disease duration, were recruited for the study. Acute stroke patients may respond differently to exercise protocol than chronic stroke patients. Also, depending upon the side, site and severity of the lesion in the brain, participants' responses to exercises may differ. Therefore, future studies may consider these factors while recruiting participants and designing their protocol. The sample size was not large, and no long-term follow-up was performed. Improvements gained with the addition of MSE and weight-shifting may be temporary and short-lived; therefore, future studies should include a long-term follow-up with large sample size. At the time of data collection, the PT examiner missed taking the participants' height, weight, and BMI values. These demographic data could have provided additional information on the association of height, weight, and BMI with stroke patients' recovery and functional progress. In the present study, balance and gait performance were measured without sophisticated equipment; future studies should use equipment such as force plates and gait analyzers to assess balance and gait performance more accurately and precisely. Future studies may explore the role of MSE in other neurological disorders where normal balance and gait are affected, e.g. cerebral palsy.

## Conclusion

A 4-week program of MSE along with weight-shifting in conjunction with CTE is more effective than a program of CTE alone in improving balance and functional gait performance in the selected stroke population. However, further randomized controlled trials should confirm these findings with large sample sizes and participants with a specific disease states like acute, subacute and chronic.

## Data Availability

The data associated with the paper are not publicly available but are available from the corresponding author on reasonable request.
